# Supplementary material for: Modelling the distribution of white matter hyperintensities due to ageing on MRI images using Bayesian inference
Source: Neuroimage. 2019 Jan 15;185:434–45. doi: 10.1016/j.neuroimage.2018.10.042 (PMC6299259; doi:10.1016/j.neuroimage.2018.10.042)
Supplement: Supplementary_material_YNIMG_15361_V3 [file mmc1.pdf]

Supplementary material: Modelling the distribution of white matter hyperintensities due to ageing on MRI images using Bayesian inference

Effect of b-spline knot spacing on algorithm results in simulated data

As a part of our algorithm development, we analysed the effect of various parameters of our algorithm on the final lesion probabilities. One of the most important parameters in our algorithm is the knot spacing in the spline model. We studied its effect and how it relates to the smoothness of kernel K used for the generation of the simulation data, by varying them independently and evaluated their combined effect on the algorithm result $\hat{\theta}$.

Figure S1 shows the effect of change in spline knot spacing and in the standard deviation of kernel K used in the ground truth estimation independently on the error values on the simulated data. For this experiment we used knot spacings of 2 and 6 voxels in our algorithm, while in ground truth generation we used a Gaussian smoothing kernel K with standard deviation values of 0.8 and 1.5. As we can observe from figure S1, the error image corresponding to the smaller knot spacing has lower MSE values of 7.27×10^{-5} and 4.83×10^{-4} for both the standard deviation values used in the smoothing kernel. On the other hand, the splines with larger knot spacing performed much worse with respect to smoother ground truth distribution (generated using standard deviation of 1.5) with the highest MSE of 1.00×10^{-3}. With the small knot spacing the splines are still able to model a smooth function and gave lower error value for both the cases. However, with a large knot spacing there is a limitation to how well it can model less smooth functions. Moreover, the MSE values are higher for the larger knot spacing for any given smoothness of ground truth distribution. Hence we chose the smaller spacing of value 2 for spline

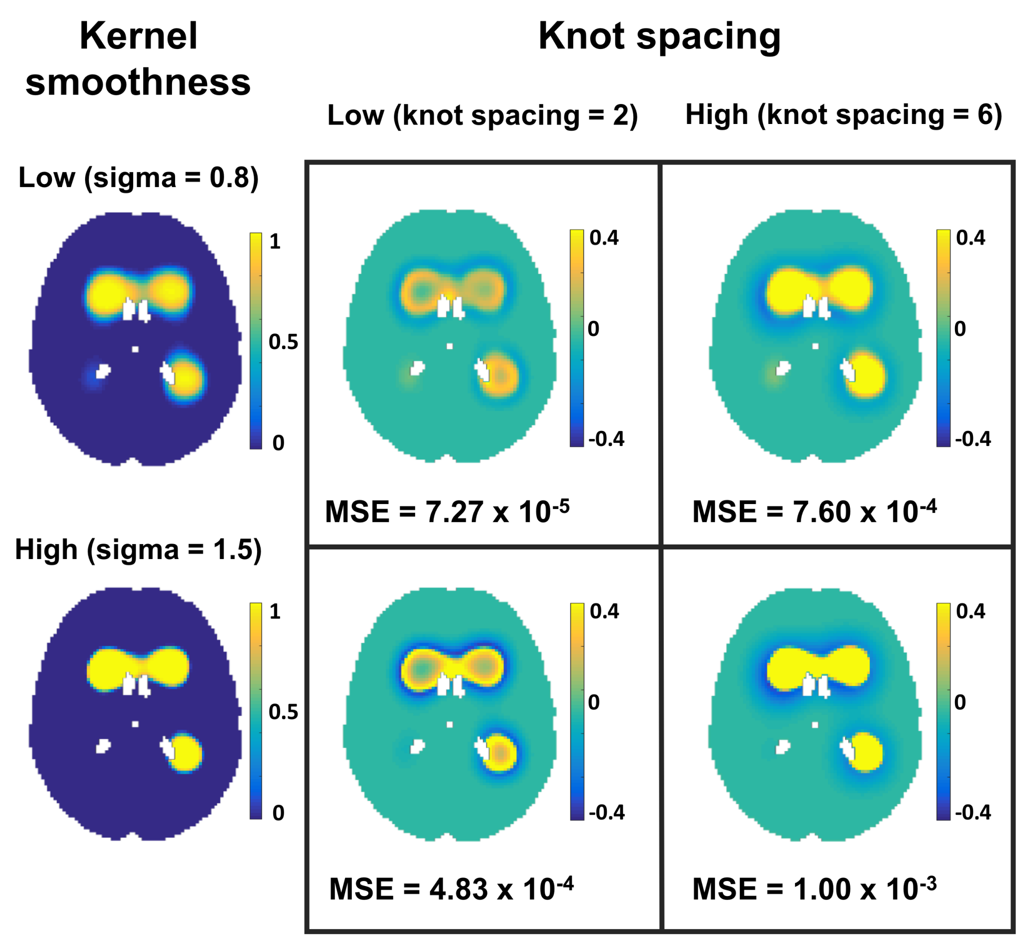

Figure S1: Comparison of error maps for various values of knot spacing in our Bayesian spline method $\hat{\theta}$ and standard deviation (sigma) values for smoothing kernel in simulation ground truth θ_{true}. Within the table, the top row shows error maps and mean-squared error (MSE) values, when θ_{true}, generated using a smaller symmetric kernel (sigma = 0.8) is compared against $\hat{\theta}$ modelled with smaller and larger b-spline knot spacing of 2 and 6 voxels respectively. Similarly, the bottom row shows error maps and MSE values for comparison of smoothing kernel K with larger symmetric kernel (sigma = 1.5) in θ_{true} against $\hat{\theta}$ with two different b-spline knot spacings.

approximation of real data.

Modelling the distribution of WMH with respect to Montreal cognitive assessment (MoCA) score

For the main experiments in this paper we considered age as our parameter of interest. However, our modelling algorithm can work with any continuous parameter of interest. For example, we also modelled the distribution of WMH on OXVASC

data, considering Montreal Cognitive Assessment (MoCA) score as factor of interest. In fact, apart from age, cognitive impairment is another parametric factor that has been widely associated with WMH. It has been shown that MoCA score is correlated with the presence of WMH, particularly in frontal periventricular areas [1]. Hence we modelled the distribution of WMH within a subset of the OXVASC dataset with respect to the MoCA scores. We considered the 411 subjects for which the MoCA score was available (MoCA scores range 12 - 30, mean MoCA score = 26.17 ± 3.54). We grouped the subjects having MoCA scores lower than 20 (N = 22) in a single bin and grouped the rest of the subjects in the MoCA score bins of 1 to form a 4D image of dimensions $91 \times 109 \times 91 \times 12$ and applied our model.

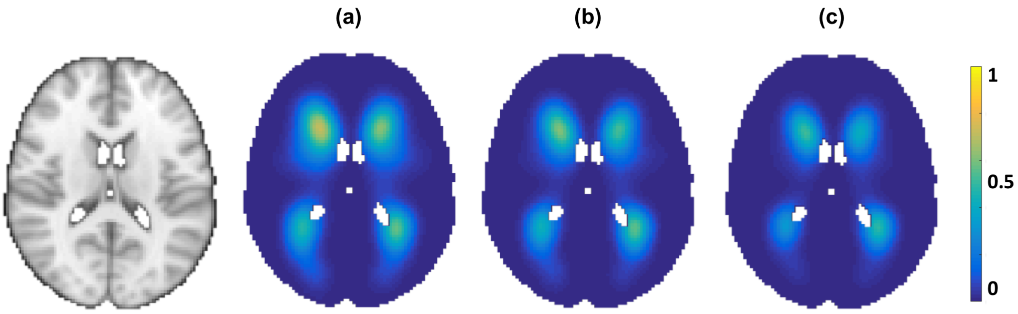

Figure S2: Population-level lesion probability map with respect to MoCA scores. The result of our Bayesian modelling method is shown for three representative cases: (a) MoCA score < 20 (severe cognitive impairment), (b) MoCA score = 25 (mild cognitive impairment) and (c) MoCA score = 30 (cognitively healthy). The MNI template is shown on the left and all the results have been shown at the slice z = 45.

Figure S2 shows the resulting population-level parametric lesion probability map obtained from our Bayesian modelling algorithm on the OXVASC data for three MoCA scores: MoCA score < 20 (severe cognitive impairment), MoCA score = 25 (mild cognitive impairment) and MoCA score = 30 (cognitively healthy subject). We observed that the population-level WMH probability values decrease with increase in the MoCA scores. This trend can also be observed in the plot of lesion probability values shown for a frontal periventricular voxel in figure S3. This lower probability of finding WMH in cognitively healthy subjects compared with the subjects having cognitive impairment is in line with the literature [1].

Effect of initialisation on algorithm results in OXVASC data

In order to test the robustness of our method, we evaluated the convergence of our algorithm by providing different starting points (initialisations) on the OXVASC data and provided the results below. Since the scope of our Bayesian modelling method is its application to WMH of presumed vascular origin, we used the smoothed

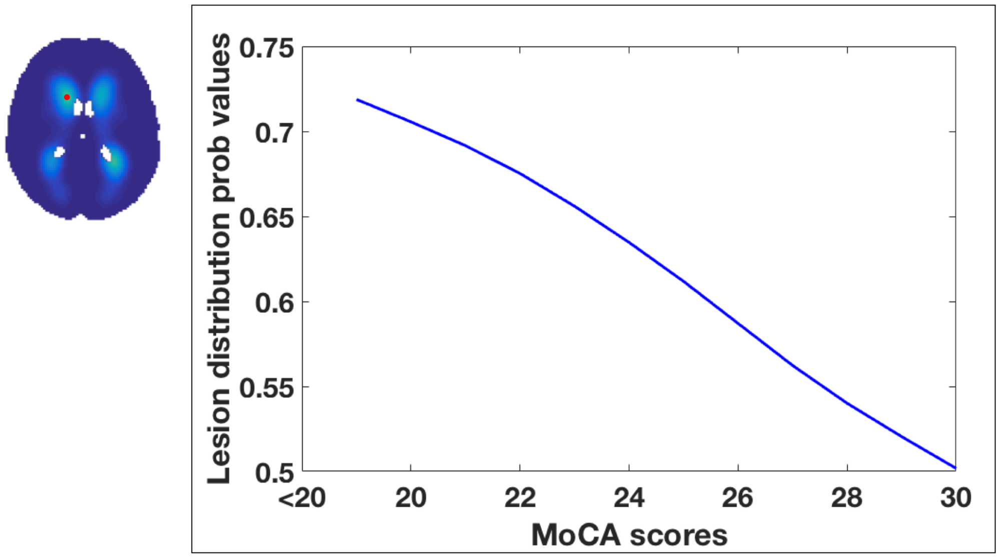

Figure S3: Plot of WMH probability values versus MoCA scores. The plot shows lesion probability values for a frontal periventricular voxel (indicated by the red dot in the image on the left).

R_i/N_i map with the specified lesion pattern as our initialisation. Hence, as sensible options for different initialisations, we introduce perturbations in the R_i, smooth the ratio R_i/N_i and provide them as initialisation.

For providing different initialisations, we modified each voxel of the R_i map by sampling a value randomly within a specific percentage of the existing voxel value. For this experiment, we considered three percentages of perturbation: 10%, 30% and 50%. For example, for a perturbation of 10% in a voxel which has an initial R_i value of 20, a value was chosen randomly in the interval [18, 22].

Figure S4 shows the results our Bayesian modelling method with different initialisations based on different percentages of perturbations. The figure shows the difference maps obtained by comparing the algorithm results obtained for different initialisations with the result for the original/unperturbed initialisation. From the figure, we can observe that for initialisations obtained by perturbing the R_i by 10% and 30%, the results of our Bayesian modelling method show very small differences with respect to our results obtained from the unperturbed R_i. The corresponding difference maps show negligible mean square difference (MSD) values of 4.43×10^{-6} and 8.92×10^{-6} (with respect to the unperturbed case) for the perturbations of 10% and 30% respectively. For comparison, these values are lower than the MSE value of 7.7×10^{-5} that we obtained in our simulation analysis for our method with respect to the ground truth distribution. The difference maps corresponding to the case of 50% perturbed initialisation gives an MSD value of 1.19×10^{-5} (which is quite close to the MSE values obtained in our simulation analysis).

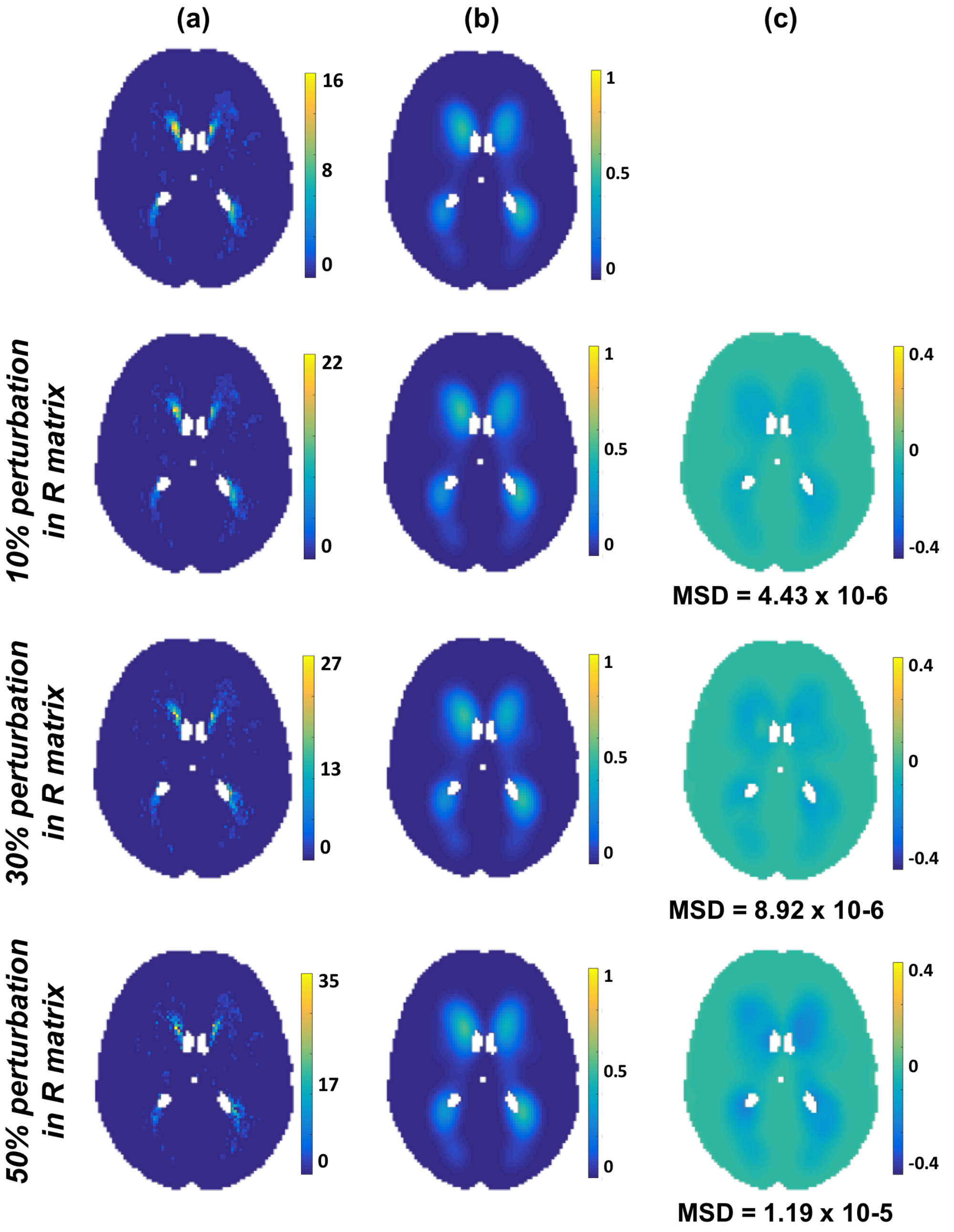

Figure S4: Results for different initialisations with various levels of perturbations. The second to fourth rows correspond to the results for different initialisation obtained with introducing 10%, 30% and 50% perturbation in the group-wise average 4D map R_i (top row, column a). (a) group-wise average 4D map R_i, (b) result of our Bayesian modelling method and (c) difference maps with respect to the result of initialisation from unperturbed R_i (top row, column b).

These additional results show that our method is quite stable and converging on very similar results as shown numerically and visually (in figure S4). For robust results, up to 30% perturbations can be tolerated by the method with changes an order of magnitude smaller than the errors obtained in our simulation data.

Effect of initial subject-level thresholds on algorithm results in OXVASC data

We evaluated the sensitivity of our Bayesian modelling method with respect to the thresholds applied on the subject-level lesion probability maps obtained from BIANCA. To this aim, we used the 474 subjects from OXVASC dataset. The subject-level lesion probability maps were obtained using BIANCA with the modalities and parameters specified in [2]. As shown in supplementary figure S4 (F) of [2], threshold values of 0.85 to 0.95 gave reasonably good similarity indices (Dice index) during BIANCA optimisation. The threshold value of 0.9 gave the maximum Dice index and hence was used for obtaining the binary lesion maps in [2] and in our work. We have now used 0.85 and 0.95 for the suggested test.

Figure S5 shows the results of the Bayesian modelling method when our subject-level lesion probability maps were thresholded at 0.85 and 0.95, compared with those obtained at the threshold of 0.9. From the figure, we can observe that both the MSD values of 1.3×10^{-4} and 1.5×10^{-4} are higher than the MSE value of 7.7×10^{-5} obtained in our simulation analysis. This is due to the fact that increasing the threshold value from 0.85 to 0.9 and from 0.9 to 0.95 results in the reduced lesion volumes (by approx. 300 mm^3 and 400 mm^3 per subject respectively) in the binary lesion maps provided as input to our algorithm.

However, the overall relationship of the lesion distribution probabilities with respect to age remains same (i.e. lesion distribution probability values increases with age) for all the thresholds as shown in figure S6.

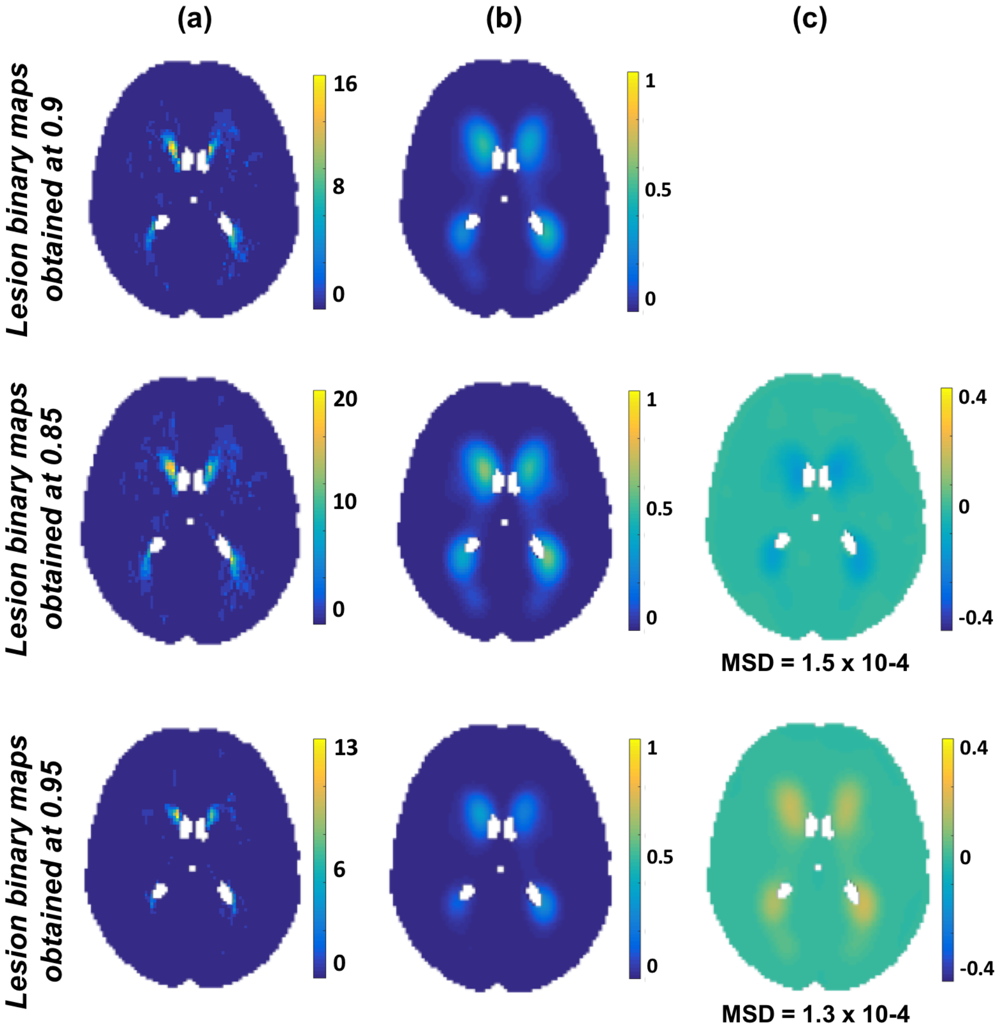

Figure S5: Results of the Bayesian modelling method when subject-level lesion binary maps are obtained using different thresholds. The rows correspond to the results for individual binary maps obtained using thresholds 0.9 (top row, used in the manuscript), 0.85 and 0.95 (middle and bottom row, respectively). (a) group-wise average 4D map R_i, (b) result of our Bayesian modelling method and (c) difference maps with respect to the result of binary maps obtained using the threshold of 0.9 (top row, column b).

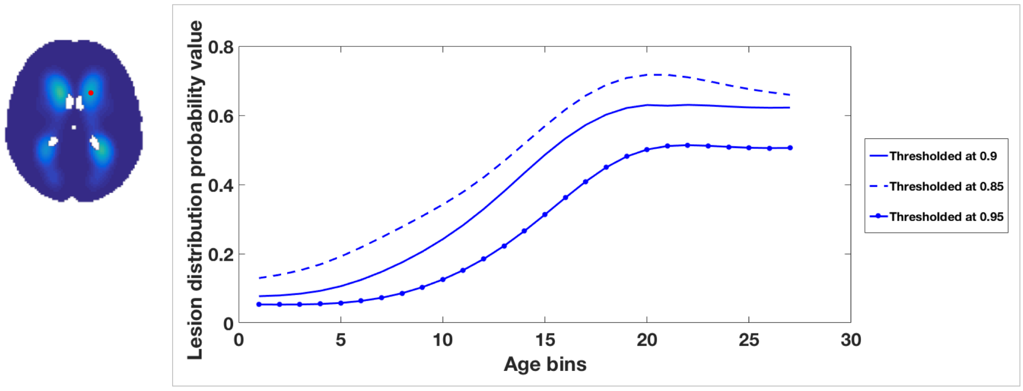

(a)

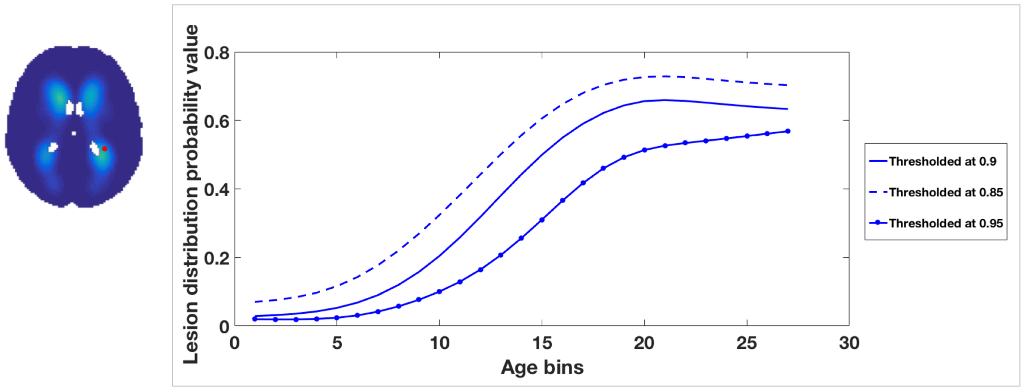

(b)

Figure S6: Lesion probability values plotted versus age for various thresholds. The plot of lesion probability values shown for (a) an anterior and (b) a posterior voxel. The red dots on the population-level lesion probability map indicate the voxel locations corresponding to the plot.

References

[1] Giovanna Zamboni, Ludovica Griffanti, Mark Jenkinson, Sara Mazzucco, Linxin Li, Wilhelm Küker, Sarah T Pendlebury, and Peter M Rothwell. White matter imaging correlates of early cognitive impairment detected by the montreal cognitive assessment after transient ischemic attack and minor stroke. *Stroke*, 48(6):1539–1547, 2017.

[2] Ludovica Griffanti, Giovanna Zamboni, Aamira Khan, Linxin Li, Guendalina Bonifacio, Ursula G Schulz, Wilhelm Kuker, Marco Battaglini, Peter M Rothwell, and Mark Jenkinson. BIANCA (brain intensity abnormality classification algorithm): a new tool for automated segmentation of white matter hyperintensities. *NeuroImage*, 2016.
